# AKU Giant Pituitary Adenoma Score: A Novel Scoring System to Predict the Outcomes of Surgery for Giant Pituitary Adenomas

**DOI:** 10.7759/cureus.29232

**Published:** 2022-09-16

**Authors:** Syed A Enam, Sanam M Ghazi, Muhammad F Raghib, Adnan Salim, Shiraz Hashmi, Fauzan Hashmi, Saad B Anis, Muhammad Bilal Tariq, Meher Angez, Shahzad Shamim, Ahsan A Khan

**Affiliations:** 1 Surgery, Aga Khan University, Karachi, PAK

**Keywords:** agpa score, surgery outcomes, tumor surgery, tumor resection, giant pituitary adenomas

## Abstract

Background

No scoring system is available to predict the extent of resection of giant pituitary adenomas (GPAs) based on magnetic resonance imaging (MRI) parameters. We developed a novel AKU Giant Pituitary Adenoma (AGPA) score and assessed the predictive ability of the scoring system concerning the extent of resection of GPAs.

Methodology

We retrospectively collected data of patients presenting with GPAs and used our scoring system to assess the surgical resection of these tumors. The Lundin-Pederson (ABC/2) method was used to calculate the pre- and post-resection tumor volume. The relationship between the extent of resection and the AGPA score was assessed using linear regression. The AGPA score considered the tumor’s extension into various planes. The maximum total score was 9.

Results

The scoring system was applied to 45 patients with GPA who underwent surgical resection. The mean resected tumor volume (%) was 82.0 ± 16.7, and the overall mean AGPA score was 4.2 ± 0.8. The pairwise correlation between the resected tumor volume and the overall AGPA scores showed a strong inverse association (r = -0.633, p < 0.001). A significant difference was detected between the estimated scores of 3 and 5 and 4 and 5 (p < 0.001).

Conclusions

AGPA score is inversely related to the extent of the tumor to be resected, which would help surgeons predict the amount of tumor resection possible as well as predict the difficulty of surgery and plan optimal preoperative patient counseling. In addition, it can predict if staging and a transcranial approach are required.

## Introduction

Pituitary adenomas make up about 10-15% of all intracranial neoplasms [1]. Giant pituitary adenoma (GPA) accounts for 5% of all pituitary adenomas and is defined as a tumor measuring 4 cm or more in diameter in its largest dimension [2]. These tumors expand by growing in the sella and extending into the suprasellar and parasellar regions [3]. Their large volume can cause symptoms of increased intracranial pressure such as headache, visual field defects, endocrinological abnormalities, and ophthalmoplegia [4]. Due to their invasiveness, such tumors can be a surgical challenge. Standard management includes magnetic resonance imaging (MRI) followed most commonly by (open or endoscopic) trans-sphenoidal and, occasionally, transcranial resection, though it is difficult to predict the amount of tumor which can be resected [5,6]. Several authors have proposed classifications for a radiological description of tumor extension and its relationship with surgical outcomes [3,5,7-10]. However, no scoring system exists to predict the degree of resection of GPA based on preoperative MRI parameters.

This study aimed to develop the AKU Giant Pituitary Adenoma (AGPA) score, a preoperative scoring system based on MRI parameters that can be used to help predict the extent of tumor resection and difficulty of surgery using the standard trans-sphenoidal approach. Such a scoring system would also help in preoperative patient counseling and their overall clinical management. In the past, pituitary adenomas have been classified without establishing correlations between tumor grading and the extent of tumor resection. Our scoring system builds upon these categorizations and, unlike previous studies, is specific for GPAs. Our scores were calculated based on MRI parameters on a scale of one to nine, with one being the minimum and nine being the maximum score. This approach will allow us to estimate the extent of resection based on the difficulty of surgery, thus enabling this scoring system to be used as a research tool as well. We hypothesized that the AGPA score has an inverse relationship with the extent of resected tumor volume and has a substantial ability to predict the extent of tumor volume to be resected in GPA patients. We reviewed our 15-year record of retrospective resected GPA surgeries and applied the AGPA score prospectively to assess the predictive ability of the scoring system for the extent of resection.

## Materials and methods

This is a retrospective study of GPAs treated at the Aga Khan University Hospital (AKUH) in Karachi, Pakistan, between January 2006 and March 2021. Patients were included in the study if the MRI scans showed a pituitary adenoma with a diameter of 4 cm or more in any plane and a confirmatory histopathological examination. We only included patients who were operated on at our center and whose MRI scans were available for review. To assess the extent of resection, postoperative MRI scans were obtained and reviewed for the purpose of this study. The MRI scans were either reviewed by two different investigators, or by the same author at two different times. Patients with previous pituitary tumor resection were excluded. A total of 45 patients were included in the study after performing the preoperative hormonal workup, endocrinological assessment, and ophthalmological assessment.

Independent variables recorded included clinical symptoms, endocrinological dysfunction, tumor dimensions on MRI, tumor volume based on the Lundin-Pederson model (ABC/2) used to measure the volume of intracranial tumors, extension into neighboring structures based on MRI, and postoperative outcomes such as hypopituitarism, improvement of preoperative symptoms, and need for re-operations or stereotactic radiosurgery [11-13]. The extent of tumor resected was grouped as follows: gross total resection (defined as no tumor visible on postoperative MRI), near-total resection (less than 10% residual tumor volume), subtotal resection (10-20% residual tumor), and partial resection (more than 20% residual tumor) [2].

AGPA score

**Figure 1:**
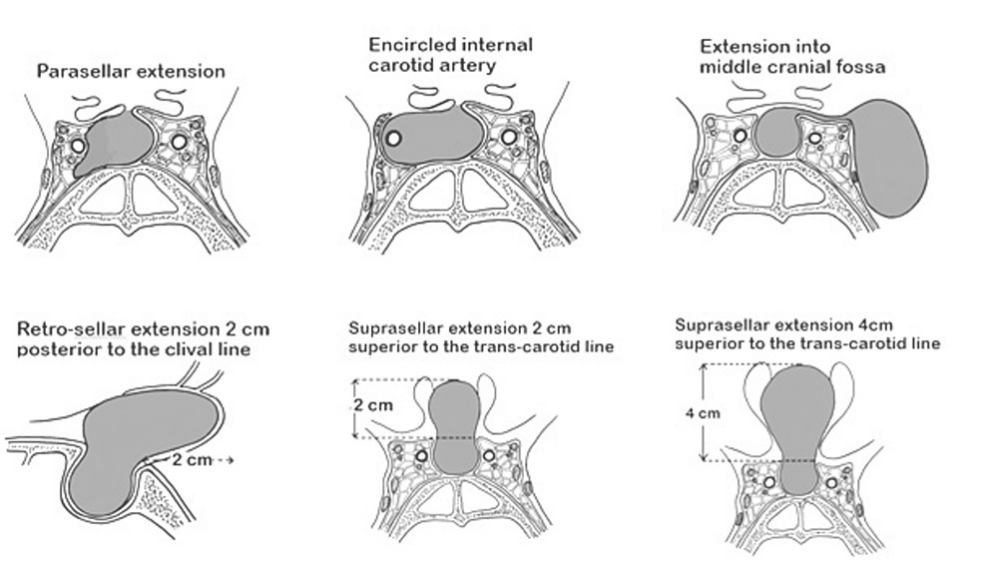
Overview of the tumor extending in various planes: parasellar extension, suprasellar extension >2 cm superior to the transcarotid line, suprasellar extension >4 cm superior to the transcarotid line, retrosellar extension 2 cm posterior to the clival line, and encasement of the cavernous internal carotid artery.

A new scoring system based on preoperative MRI findings was developed. A rigorous literature search was performed to explore diagnostic parameters complemented by anecdotal evidence to develop a novel scoring system to predict the volume of the tumor to be resected [3,7,9,10]. Points were given for tumor extension into neighboring structures that usually contributes to the complexity of the surgery. For parasellar extension and internal carotid artery completely encased by the tumor in the cavernous sinus and extension into the anterior or middle cranial fossa, each side was given one point. For retrosellar extension 2 cm posterior to the clival line, defined as the line extending from the posterior clinoid to the odontoid tip, one point was given. For suprasellar extension 2 cm superior to the transcarotid line, defined as the line joining two cavernous carotids in the coronal plane, one point was given. For extension over 4 cm, one more point was given. One point was given if the tumor extended into either the anterior or middle cranial fossa. If the tumor extended into two or more cranial fossae, two points were given. These points were added for a total of 9 points (Table 1 and Figure 1).

**Table 1:** Overview of AGPA scoring system.

Tumor extension	Points given on the score
Parasellar extension (1)	1 + 1 (one point for extension into each side)
Encircled internal carotid artery (2)	1 + 1 (one point for each carotid that has been encircled)
Extension into the anterior or middle cranial fossa (3)	1 + 1 (one point for each fossa involved for a maximum score of two)
Suprasellar extension 2 cm superior to the transcarotid line (4)	1
Suprasellar extension 4 cm superior to the trans-carotid line (4)	1
Retrosellar extension 2 cm posterior to the clival line	1
Maximum score possible	9

Analysis

Data were analyzed using Stata (version 16.1, StataCorp LLC, College Station, TX, USA). The normality of the continuous variable was assessed by the Shapiro-Wilk test, mean ± standard deviation (SD), or median and interquartile ranges (IQRs) reported where appropriate. Categorical variables were presented as frequency and percentages, the mean of resected tumor (%) across AGPA scores was assessed by one-way ANOVA, and the post hoc Bonferroni test was applied to assess the group differences. Pairwise correlation coefficients were computed to assess multicollinearity. Univariate and multivariable linear regression was conducted using a standardized approach to get parsimonious prediction models. The correlation between the percentage of resected tumor volume and the percentage of residual tumor volume was assessed using linear regression. A p-value of <0.05 was set as significant.

## Results

A total of 45 patients with GPA were analyzed. The study population comprised 84.4% males, and the mean age of the study population was 45.6 ± 13.3 years. The most frequent symptoms the patients presented with included visual field deterioration (93.3%), followed by visual deterioration (86.7%) and headaches (44.4%). Other common symptoms were change in mentation (15.6%), ophthalmoplegia (6.7%), seizures (4.4%), and decreased or loss of libido (4.4%). The most common tumor in the study group was the non-secretory type of tumor (84.4%), followed by prolactinoma (11.1%) and growth hormone-secreting adenoma (4.4%).

The median preoperative tumor volume was 24 cm^3^ (IQR = 18.1, 36.9) and reduced up to 3.8 cm^3^ (IQR = 1.2, 8.3) after resection. The microscopic trans-sphenoidal approach was adopted in more than half of the study population (53.3%), followed by the microscopic endoscopic-assisted approach (20.0%), transcranial (15.6%), and endoscopic endo-nasal (11.1%). The mean resected tumor volume was 82.0% ± 16.7, and the mean residual volume was 18.0% ± 16.7. The overall mean AGPA score was 4.2 ± 0.8 (Table 2). Gender-stratified mean AGPA score and preoperative tumor size were comparable in both genders; the mean AGPA score was 4.2 ± 0.8 vs. 4.43 ± 0.5 (p = 0.523), and preoperative tumor size was 43.3 ± 65.8 vs. 36.79 ± 25.5 (p = 0.799). However, the resected tumor volume (%) showed a higher trend in males (84.0 ± 12.8 vs. 71.51 ± 29.7). Nevertheless, the difference did not attain statistical significance (p = 0.070).

**Table 2:** Baseline demographics and clinical and postoperative characteristics of the study population (n = 45). SD: standard deviation; IQR: interquartile range; AGPA: AKU Giant Pituitary Adenoma

Variables	Mean ± SD/n (%)
Age (years)	45.6 ± 13.3
Gender	
Male	38 (84.4)
Female	7 (15.6)
Signs and symptoms	
Visual field deterioration	42 (93.3)
Visual deterioration	39 (86.7)
Headache	20 (44.4)
Change in mentation	7 (15.6)
Ophthalmoplegia	3 (6.7)
Seizure	2 (4.4)
Low/loss of Libido	2 (4.4)
Type of tumor	
Non-secretory	38 (84.4)
Prolactinoma	5 (11.1)
Growth hormone-secreting	2 (4.4)
Preoperative tumor volume (cm^3^) [Median (IQR)]	24 (18.1, 36.9)
AGPA score	4.2 ± 0.8
Surgical approach	
Microscopic trans-sphenoidal	24 (53.3)
Microscopic endoscopic-assisted	9 (20.0)
Transcranial	7 (15.6)
Endoscopic endo-nasal	5 (11.1)
Resected tumor volume (%)	82.0 ± 16.7
Postoperative tumor volume (cm^3^) [Median (IQR)]	3.8 (1.2, 8.3)
Residual tumor volume (%)	18.0 ± 16.7

Pairwise correlation between the dependent (resection volume) and main explanatory variable (overall AGPA scores) showed a strong inverse association (r = -0.633, p < 0.001). However, the association between age and preoperative tumor size fell in the weaker ranges (Table 3).

**Table 3:** Pairwise correlation matrix of resected tumor volume, overall AGPA score, age, and preoperative tumor size. AGPA: AKU Giant Pituitary Adenoma

Variables	Resected tumor volume (%) r* (p-value)	Total AGPA score r* (p-value)	Age in years r* (p-value)
Overall AGPA score	-0.633 (<0.001)	-	-
Age in years	0.035 (0.821)	0.084 (0.585)	-
Preoperative tumor volume	0.152 (0.321)	0.138 (0.367)	0.099 (0.558)

**Figure 2:**
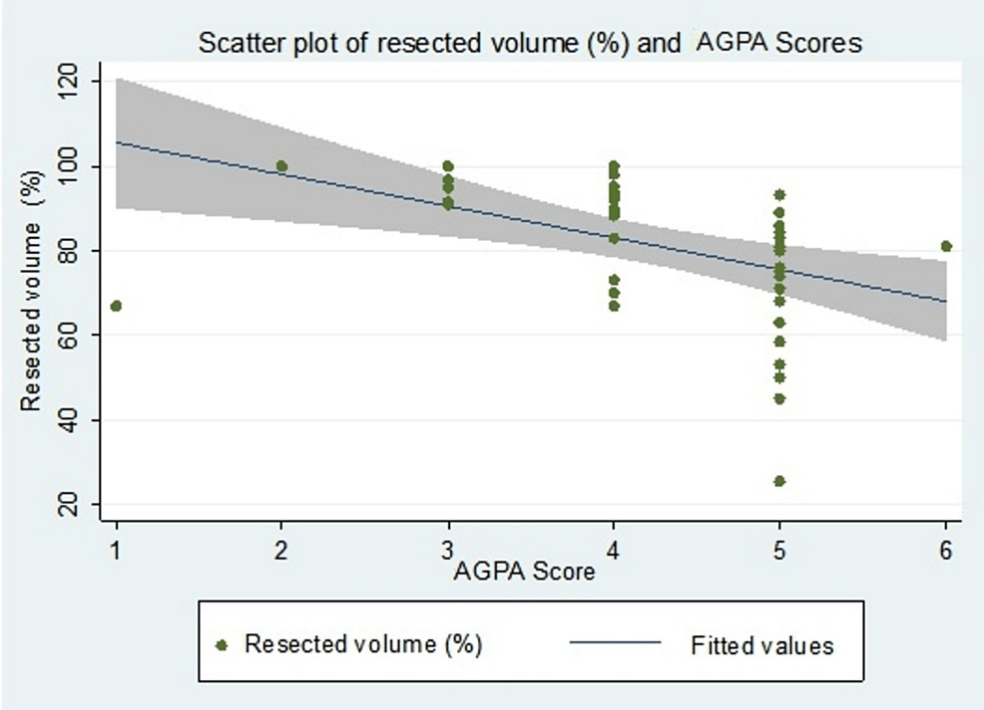
Scatter plot showing the correlation of resected volume and AGPA score with a fitted regression line with 95% confidence intervals. AGPA: AKU Giant Pituitary Adenoma

An inverse relationship between resected tumor volume and AGPA scores was evident; as the AGPA scores progress, resection volume regresses. A significant difference was detected between estimated scores of 3 and 5 and 4 and 5 (p < 0.001); however, no significant difference was observed between scores 2-3 and 4 (p = 0.638) (Table 4). A similar association can be visualized in the scatter plot (Figure 2).

**Table 4:** Mean tumor volume resected (%) by AGPA scores (n = 45). *: total score ranged from 0 to 9; however, no patient scored 0 or 1 and >5; **: one-way ANOVA. AGPA: AKU Giant Pituitary Adenoma; ANOVA: analysis of variance

AGPA score*	n	Resected volume (%)	Difference of score	Mean difference (%)	P-value**
2–3	7	96.3 ± 3.9	4 and 3	-7.2 ± 5.7	0.638
4	19	89.1 ± 9.4	5 and 3	-26.7 ± 5.7	<0.001
5	19	69.7 ± 17.2	5 and 4	-19.4 ± 4.2	<0.001

At the univariate level, a significant inverse association was determined between overall resected tumor volume and AGPA scores. The significance persists after adjusting for age, gender, and preoperative tumor size (Table 5, Model 1). The estimated β coefficient for AGPA scores was -14.728 (95% confidence interval (CI) = -19.182, -10.274), indicating that with one unit increase in AGPA score, an average of 15% decrease in resection volume can be predicted (p < 0.001). Similarly, the association of preoperative tumor size and resected tumor were positively associated; with every one unit increase in preoperative tumor size, an average of 16% tumor resection can be predicted (p = 0.012). The female gender showed an inverse trend compare to the male gender; the β coefficient was -9.161 (95% CI = -25.045, 6.723), indicating that the tumor volume in females is 9% less likely to be resected compared to males; however, this association did not attain statistical significance (p = 0.250). R square was 0.534 showing that a 53% variability can be explained by this model (p < 0.001) (Table 5).

**Table 5:** Univariate and multivariable linear regression Model-1 showing correlation of overall resected Tumor Volume (%) and AGPA Scores. **: multivariable estimates adjusted for age, gender and preoperative tumor volume, R^2^ = 0.534; F-statistic = F (5, 38), 12.24; p < 0.001. CI: confidence interval; AGPA: AKU Giant Pituitary Adenoma

Resected tumor volume (%)	Crude β coefficient (95% CI)	P-value	**Adjusted β coefficient (95% CI)	P-value
AGPA score	-14.697 (-19.952, -9.443)	<0.001	-14.728 (-19.182, -10.274)	<0.001
Age	0.111 (-0.197, 0.418)	0.472	0.052 (-0.242, 0.346)	0.724
Gender	0.111 (-0.197, 0.418)	0.247	-9.161 (-25.045, 6.723)	0.250
Preoperative volume	0.067 (0.015, 0.118)	0.031	0.157 (0.036, 0.278)	0.012

Model 2 regression model was also built considering AGPA score of 2-3 as the reference, and relative coefficients of high scores were estimated. At the univariate level, a consistent inverse correlation between resected tumor volume and AGPA scores of 4 and 5 with reference to scores of 2-3 was determined. The significance persists after adjusting for age and preoperative tumor volume at both the scores of 4 and 5, except for a score of 4 where the association of gender did not reach the threshold of significance (p = 0.149) (Table 6). At the multivariable level (Model 2), AGPA score of 4, the β coefficient was -7.481 (95% CI = -14.327, -0.635), showing that the average resection volume was further decreased by 7.5% with reference to the score of 2-3 (p = 0.033), while at a score of 5, the estimated β coefficient was -26.783 (95% CI = -33.758, -19.809), indicating an average of 27% tumor volume can be further resected with reference to the scoring of 2-3 (p < 0.001). Age and preoperative tumor volume were positively associated with the outcome. An estimated β coefficient for age was 0.051 (95% CI = -0.223, 0.326), showing that with a one year increase in age, a 5% volume can be resected; however, the p-value was insignificant (0.706). Likewise, the β coefficient estimated for baseline tumor size was 0.136 (95% CI = 0.004, 0.268) eliciting that with every one unit increase in tumor size, 14% tumor can be resected (p = 0.044). The adjusted β for the female gender was -10.369 (95% CI = -25.386, 4.649), which specifies females were 10% less likely to be resected compared to males; however, this association did not attain statistical significance (p = 0.170). The estimated R square was 0.561, explaining 56% of variability by Model 2 (p < 0.001) (Table 6).

**Table 6:** Univariate and multivariable linear regression Model 2 showing the correlation of resected tumor volume and AGPA scores of 4 and 5 with reference to the score of 2-3. *: with referee to the AGPA score of 2-3; **: multivariable estimates adjusted for age, gender, and preoperative tumor size; R^2^ = 0.561, F(5, 38), 3.31, and p < 0.001. CI: confidence interval; AGPA: AKU Giant Pituitary Adenoma

Resected tumor volume (%)	Crude β coefficient (95% CI)	P-value	**Adjusted β coefficient (95% CI)	P-value
AGPA score 4*	-7.019 (-12.795, -1.243)	0.018	-7.481 (-14.327, -0.635)	0.033
AGPA score 5*	-26.938 (-35.538, -18.337)	<0.001	-26.783 (-33.758, -19.809)	<0.001
Age	0.132 (-0.165, 0.429)	0.375	0.051 (-0.223, 0.326)	0.706
AGPA score 4*	-4.836 (-11.480, 1.809)	0.149	-	-
AGPA score 5*	-24.881 (-31.849, -17.914)	<0.001	-	-
Gender (female)	11.4 (-3.976, 26.777)	0.142	-10.369 (-25.386, 4.649)	0.170
AGPA score 4*	-8.434 (-13.738, -3.131)	0.003	-	-
AGPA score 5*	-28.361 (-37.004, -19.718)	<0.001	-	-
Preoperative volume	0.065 (0.020, 0.110)	0.006	0.136 (0.004, 0.268)	0.044

## Discussion

We attempted to develop a standardized scoring system that can be used to predict the volume of the tumor that can possibly be resected and predict the difficulty of surgery. The AGPA score is a first-of-its-kind scoring system that incorporates the extension of the tumor in multiple planes. In the scoring system we developed, AGPA, a maximum score of 9 is possible, and its correlation with the amount of resection is also highlighted. An inverse trend was evident between resected tumor volume and AGPA scores; as the AGPA scores progress, resection volume decreases. This was according to our expectation because the greater the tumor size, the more difficult it becomes to resect it. There was no correlation between increasing tumor size and the extent of resection while the relationship between AGPA score and the extent of resection was significant, backing our claim that the direction of spread is more important than the overall tumor size. In addition, as the score increased, the incidence of recurrence increased, along with the increased need for redo surgery or stereotactic radiosurgery. This was probably due to inadequate resection of the initial tumor. While more data for tumors that are scored 7 or higher is needed, we hypothesize that a score of 7 or higher can be used as an indication for transcranial resection.

Multiple cut-offs have been used to define GPAs. However, the most common is the tumor size equal to or greater than 4 cm in its largest dimension [2,5,12,14]. Due to their size, GPAs can compress multiple local structures resulting in various symptoms. Surgical resection is the standard of treatment, with trans-sphenoidal being the most common approach, followed by the transcranial approach. However, considering the invasion of giant adenomas, size is not the only variable on which the degree of resection depends as invasion in different directions makes it difficult for complete resection using the trans-sphenoidal approach.

Multiple studies have been conducted on the resection rates of GPA. The extent of resection varies from 14.7% and 22.2% gross total resection reported by Mortini et al. and Wang et al. to 46.5% and 67% reported by Nakao et al. and Guo et al. [2,5,6,10,12,15,16]. However, it must be noted that Guo et al. used a transcranial approach in all patients. The inconsistency in the extent of resection in our data may have been due to differences in the surgeon’s experience with GPAs, the difference in the size and invasion of the tumor, and differences in evaluating the tumor size and residual tumor volume.

The first attempt at classifying pituitary adenomas was made by Hardy et al. in 1976 which was expanded on by Wilson et al. in 1984. This classification divided pituitary adenomas based on their size and extension [7,9]. This was followed by Knosp et al. who developed a grading system that was helpful in assessing cavernous sinus involvement [3]. This grading system was limited by the fact that it took only cavernous involvement into account. Because giant tumors can spread extensively, the grading system by Knosp et al. grading may not be sufficient. The SIPAP classification combined these two classifications to develop a grading system that considered extension in all directions [17]. While it did a good job at describing the spread of the tumor to give an idea to the surgeon as to how difficult the surgery may be, the six-digit numbering system can make it cumbersome to use, and no clear correlation between grading and extent of resection has been shown. The AGPA score, however, is user-friendly and easy to use and interpret by the surgeon. Additionally, none of these scoring systems were specific for GPAs. Goel et al. developed a grading system for GPAs; grades I-IV were described based on the extension of the tumor. These grades were helpful in predicting the amount of tumor resection possible [5]. However, the grading was still restricted to spread in limited directions and by the large difference in total resection between grades I and II. Sinha et al. also grouped giant pituitary tumors based on the direction of spread [10].

In this study, we developed a new scoring system by expanding on these divisions by grouping tumors depending on the tumor extension, size, and invasion into different directions along with the degree of difficulty in approaching those direction systems. The score was calculated based on MRI findings on a scale of one to nine, with one being the minimum and nine being the maximum score. Such a strategy offers a reasonable opportunity to estimate the extent of resection and difficulty of surgery as well as compares the invasion of the tumor, which can also be used as a research tool. Various classifications of pituitary adenoma have been described earlier and, although previous studies developed a grading system for GPAs, they covered specific areas of tumor invasion. This resulted in a large discrepancy between grades for the amount of tumor resected [5]. We have introduced a scoring system that considers extensive invasion into surrounding anatomical structure and aims to provide an idea to the surgeon of the resection possible. AGPA tool comprised a total score of 9; however, because no case beyond the score of 5 could be achieved, we were unable to infer the association between the resected volume on higher AGPA scores with the outcomes. The idea of developing the AGPA score emerged late and the score was calculated on retrospective chart review with a single institute data that has its own inherent design issue of missing data and generalizability. We were unable to analyze all patients with pre and postoperative MRI data and several cases were excluded which could introduce a selection bias and result in the underestimation of the magnitude of the outcome. Nevertheless, the findings provide us with a direction and warrant a multicenter prospective observational study to verify and validate our findings. We believe this is the first study that may provide the basis to roll out this novel idea on a larger scale.

## Conclusions

We introduced a nine-point scoring system that can be used to predict the extent of resection based on the volume of the tumor, invasion of the tumor, and the direction of tumor spread. The AGPA score is a reliable method that has the ability to predict the extent of the tumor to be resected. The score is inversely related to the extent of tumor resected, which can help surgeons in predicting the amount of tumor resection possible, as well as in predicting the difficulty of surgery and planning optimal preoperative patient counseling. Moreover, it can help predict if staging and transcranial approach is required.
